# Estimation of sexual behavior in the 18-to-24-years-old Iranian youth based on a crosswise model study

**DOI:** 10.1186/1756-0500-7-28

**Published:** 2014-01-13

**Authors:** Katayon Vakilian, Seyed Abbas Mousavi, Afsaneh Keramat

**Affiliations:** 1 Reproductive health, School nursing midwifery, Arak University of Medical sciences, Arak, Iran; 2 Psychiatric, Research Center of Psychiatry, Golestan University of Medical Sciences, Golestan, Iran; 3 Reproductive health, School nursing midwifery, Shahroud University of Medical Sciences, Hafte Tir Square, Shahroud, Iran

**Keywords:** Crosswise model Iran, Sexual behavior, Youth

## Abstract

**Background:**

In many countries, negative social attitude towards sensitive issues such as sexual behavior has resulted in false and invalid data concerning this issue.

This is an analytical cross-sectional study, in which a total number of 1500 single students from universities of Shahroud City were sampled using a multi stage technique. The students were assured that their information disclosed for the researcher will be treated as private and confidential. The results were analyzed using crosswise model, Crosswise Regression, *T*-test and Chi-square tests.

**Findings:**

It seems that the prevalence of sexual behavior among Iranian youth is 41% (CI = 36-53).

**Conclusion:**

Findings showed that estimation sexual relationship in Iranian single youth is high. Thus, devising training models according to the Islamic-Iranian culture is necessary in order to prevent risky sexual behavior.

## Background

Adolescence is a stage of life through which a person enters adulthood. For some people, it means the beginning of sexual behaviors. Sexual relationship (intercourse) may have adverse consequences such as early pregnancies, early parenting and venereal diseases such as AIDS [1]. Iran is a young country with around 60% of population below the age of 25 and around 50% below the age of 20 [2]. In this stage of life, important behavioral patterns are formed which may affect the entire life of a person. Narcotics consumption and improper sexual behaviors begin from this stage [3,4]. As it can be claimed that AIDS in 2010 is the main problem of the youth and about more than 80% of such patients live in the developing countries such as sub Sahara. About 50% of the HIV-infected patients are 15-to-24 years olds [5].

In Iran, the third wave of AIDS, namely transmission through sexual relationship, is rising.

According to the data collected by universities of medical sciences and health services up to December 21, 2011, a number of 23,902 people have been diagnosed with HIV/AIDS in the country, 91% of which were male and 9% were female. Also, In 2012, a total number of 24,290 people were diagnosed with HIV in the country, 90.8% of which were male and 9.2% were female [6,7]. There are few studies about the reproductive and sexual health status of Iranian male and female adolescents because it is a sensitive issue in Iran [4].

By sensitive study is meant that the result of study may have serious consequences for researcher, participants and communities that the participants belong to. Research on sensitive issues has special limitations such as political, ethical, cultural and other limitations. Also, the common methods that researchers use in such areas face special challenges such as refusal to answer to questions (non-response bias) and negative social attitudes [8].

Hence, researchers have made a lot of efforts on how to ask sensitive questions. Many researchers have used indirect question methods to study sensitive issues. Warner, for example, used randomized response (RR) models [9]. Considering the problems of this method such as the need for randomized response tool (coin and the like), being costly and little cooperation of interviewee, non randomized response (NRR) models such as triangular and crosswise models with two-choice question (one sensitive and another non sensitive) have been given special attention [8]. The present study is the first crosswise model study on sexual behavior of the youth in Iran. We focused on this model because it can give us more accurate information on the prevalence of sexual behavior among the youth and eliminate the biases relating to sensitive questions.

## Methods

The present research is sectional-analytical study and a section of dissertation that documents (1,2) brought out in Additional files 1 and 2. In this research, sexual behavior of young students was studied in the period between October 2011 and March 2012 in the universities of Shahroud City, including University of Medical Sciences, Islamic-Azad University, Payam-e Noor University, University of Applied Science and Technology (two universities) and non-profit higher education institution. For taking suitable sample size, we considered prevalence 50% (p = 50%), given the use of cluster sampling (design effect = 2 and response rate = 80%), taking into consideration the first type error equal to 5% (α = 5%), a number of 1000 students would be included in the research. Because of taking crosswise method, however, the number of samples increased by 50% and samples were included in the research in the form of 75 clusters each consisting of 20 students. One limitation of this study was the lack of cooperation a university in sampling collection.

In crosswise model, participants are asked two questions. Rather than answering to each question separately, they answer both questions at once. There are two choices A and B below the questions. Participants tick the square A if their answer to both questions is similar (both Yes or both No) and square B if their answer to one question is Yes and to the other question is No. The first question is completely irrelevant to the second question and it is impossible for researcher to access to its answer. In this study, the prevalence of non-sensitive question in the society was estimated to be 24% according to the list of people in Civil Status Registration Organization whose name was Ali or Mohammad or a combination of both. The non-sensitive question was “do you have any friend or relative named Ali or Mohammad?” and the sensitive question was “have you ever had sexual relationship (intercourse)?”

Sampling was performed by multi-stage method. In this method, first each university in the city of Shahroud was considered as a category and the number of clusters was determined based on the number of students in each university. Considering that each cluster approximately equals a classroom (20-30 students) in each university, after randomly choosing the clusters from among classes of each university, all members of the class (cluster), both female and male, were included in the research. Although we didn’t have limitation sampling by marred participant, only 11.6% participants have been engaged. In Iranian culture usually engaged individuals don’t have any intercourse until they hold formal marriage ceremony. For the purpose of sampling, first all universities at the city of Shahroud and the number of their students were identified. Since the number of students of universities varied between 400 and 9000, the universities with higher number of students were allocated more clusters. Students were involved in the research after getting permission from universities and prior notification of education departments as well as instructors.

In each university, after getting written permission of universities, making necessary coordination with education department and receiving the list of classes, some classes of that day were randomly selected. Then, the objective of research was notified to the concerned instructor and permission was obtained from them to allocate the last 15 minutes of the class to filling the research questionnaires. Having entered the class, the researcher introduced himself to the students, declared the objective and necessity of the research and asked them to participate voluntarily in the research, ensuring that the data obtained from them would never be disclosed to others. Also, the name of students, universities and fields were not written in the questionnaires. The questionnaires were distributed between the students in the last 15 minutes of the class and were received when completed. Response rate this study was 97%.

## Findings

The study has been confirmed by the Ethics Committee University of Medical Sciences, Islamic-Azad University, Payam-e Noor University, University of Applied Science and Technology (two universities) and non-profit higher education institution and the written permission for sampling has been obtained from all universities involved.

Frequency demographic variables in participants were included in Table 1. According to the results achieved by this research, the prevalence of sexual relationship in girls was 39%, Cl = 33-46, and in boys was 45%, Cl = 36-46. Total prevalence of sexual relationship was 41%, Cl = 36-53.

**Table 1:** Frequency demographic variables in participants

**N(%)**	**Variables**	
535(36.1)	Boys	Sex
919(62.1)	Girls	
27(1.8)	No response	
167(11.6)	Not married but engaged	Marital status
867(60.3)	18-21	Age
571(39.7)	22-24	
1272(91.5)	Urban	Place of residence
118(8,5)	Rural	
525(36.1)	Pass life skill course	
477(32.8)	Pass family planning course	
99(6.9)	Never	Taking part in religious places
379(26.3)	Seldom	
619(42.9)	Sometimes	
346(24)	Often	
288(19.8)	No	Using violence materials
1166(80.2)	Yes	
85(6.2)	Yes	Decision to escape home
1370(93.8)	No	

The results in Table 2 revealed that 290 girls (31.6%) and 160 boys (29.9%) watched pornography movies and 94 girls (10.2%) and 51 boys (9.5%) watched satellite programs. 22.1% of girls and 15.9% of boys had not been subject to pornography media, which showed a meaningful difference between males and females (p = 0.001). Cross wise logistic regression in Table 3 showed that there is a meaningful relation between age and sexual behavior, as sexual behavior in the age 21 is 3.7 times more than that in the age 18 (p = 0.012). However, it didn’t apply to other variables of the research such as sex, going away from home, using pornography materials, familiarity with life skills and living in city or village.

**Table 2:** Frequency of the type of pornography materials used in the past six month by participants

**Type of media**	**Girls**	**Boys**	**Total**	**P value**
	**N(%)**	**N(%)**	**N(%)**	
Book	52(5.7)	21(3.9)	73(5.0)	0.001
Film	290(31.6)	160(29.9)	450(30.9)	
Picture	78(8.5)	31(5.8)	109(7.5)	
Satellite	94(10.2)	51(9.5)	145(10.0)	
Other	5(0.5)	6(1.1)	11(0.8)	
A combination of the above materials	197(21.4)	181(33.8)	378(26.0)	
No use	203(22.1)	85(15.9)	288(19.8)	
Total	919(100.0)	535(100.0)	1454(100.0)	

**Table 3:** Cross wise logistic regression between some of factors and sexual behavior in participants

**Sexual relationship (dependent variable)**	**Odds ratio**	**Confidence interval (CI)**	**P value**
Sex	0.78	0.509-1.22	0.28
Going away from home (who didn’t leave to who left)	0.98	0.29-3.27	0.98
Age 21 compared with age 18	3.7	1.32-10.50	0.01
Life skills (Who passed to didn’t pass)	0.98	0.29-3.27	0.98
Urban compared with rural	1.20	0.52-2.78	0.66
Pornography materials (Those who watched pornographic materials to those who didn’t watch)	0.91	0.62-1.32	0.64

## Discussion

According to the results of this research, the prevalence of sexual relationship among the students is 41%. According to the research carried out by Mohammadi et al., 56% of adolescents had not experienced sexual relationship. 27.7% had experienced sexual contact [4]. The low prevalence in this study to our study is attributed to age and sex, was conducted in high school boys. By conducting a qualitative study on students, Mousavi et al. concluded that many factors contribute to friendships before marriage, e.g. person’s attitude, feeling of loneliness, surrounding atmosphere including university environment, and mass media which present western culture [10]. Also, a research on 1761000 Muslim Indian young people between the age 15 and 19 revealed that 15% of them had experienced sexual relationship, with 8% having sex during the past year [11]. According to a research conducted by the Center for Controlling Diseases in 2008, 47.8% of high school students had experienced sexual relationship, with 7.1% experiencing their first sex before the age of 13 [12]. According to studies, Asian and American youth are more conservative and start having sex in higher ages [13]. In Iran, likewise, the governing norms and values of the society in connection with avoidance of sexual relationship before marriage has caused most of the young people to control premarital sex [14,15]. However, there is a tendency to violate these norms. In this study, there was a meaningful relation between age and sexual relationship, as sexual relationship in the age of 21 was 3.7 times more than that in the age of 18. According to the results of this study, girls and boys had watched pornography films during the past 6 months and watched satellite programs. According to studies access to western media may result in gradual change of the norms in the societies. In 2006, a research conducted in the US revealed that the media meaningfully influences the sexual behaviors of adolescents. Considering the age, race, sex and economic status, the results indicated that the media contributes to the intention of sexual behavior by 13% [16]. Insufficient sexual health education and failure in AIDS prevention are the major problems identified in these countries. Cultural taboo, economic circumstances, sexual health problems, penetration of religion and other ethical issues are among national policy barriers in this connection [17]. In addition, such factors as fast growth of population, modernism, industrialization urbanization and penetration of foreign cultures contribute to increase of risky sexual behaviors [18].

## Conclusion

According to the results, it seems that sexual behavior is prevalent among the youth in the end of their adolescence. Therefore, considering that Iran has a young population, it is essential to prepare efficient and comprehensive programs on reproductive health by using an Iranian and Islamic model*.* These programs have to be carried out on the basis of exhaustive studies, with an emphasis on the factors contributing to sexual behaviors among girls and boys, and efficient solutions have to be adopted to control them. In view of the fact that social norms and culture of the society contribute to the avoidance of sexual relationship before marriage and influence sexual behaviors of the youth, efficient patterns need to be provided in accordance with Iran and Islamic culture and social factors, which are the main barrier to harmful behaviors such as sexual relationship before marriage, in order to prevent risky behaviors.

## Abbreviations

CI: Confidence interval.

## Competing interests

The authors declare that they have no competing interests.

## Authors’ contributions

This essay was prepared by collective efforts of all the authors. KV wrote the primary draft. SAM revised the primary draft and AK checked the English translation of the essay. This essay is part of Doctorate Degree Thesis, which was accepted in ethic committee (cod 890/08) and financed by Research Management of Shahroud University of Medical Sciences. Since the present research involves university students only, it is recommended that a similar research be made on public youth. All authors read and approved the final manuscript.

## Supplementary Material

Additional file 1 Questionnaire. Click here for file

Additional file 2 Questionnaire research project. Click here for file

## References

[B1] Haglund K Recommendations for sexuality education for early adolescents J Obstet Gyncol Neonatal Nurs 2006 35 3 369 375 10.1111/j.1552-6909.2006.00048.x 16700686

[B2] History of production of population data http://www.amar.org.ir/Default.aspx?tabid=339 agentType=ViewType&PropertyTypeID=119

[B3] Gutierrez J-P Bertozzi SM Conde-Glez CJ Sanchez-Aleman MA Risk behaviors of 15–21 year olds in Mexico lead to a high prevalence of sexually transmitted infections: results of a survey in disadvantaged urban areas BMC Public Health 2006 6 49 10.1186/1471-2458-6-49 16504147 PMC1409781

[B4] Mohammadi MR Mohammad K Farahani FKA Alikhani S Zare M Tehrani FR Reproductive knowledge, attitudes and behavior among adolescent males in Tehran, Iran Int Fam Plan Perspect 2006 32 1 35 44 10.1363/3203506 16723300

[B5] Fact sheet: adolescents, young people and HIV http://www.unaids/contentassets/documents/factsheet/2012/20120417_FS_adolescentsyoungpeoplehiv_en.pdf

[B6] Tedade mobtalayan be AIDS dar Iran http://www.farsnews.com/newstext.php?nn=13920208000526(persian)23/april/2013

[B7] Afzayesh 20% ebtela be AIDS e jensi dar keshwar 2010 Persian http://ircha.tums.ac.ir/(prsian site)

[B8] Yu J-W Tian G-L Tang M-L Two new models for survey sampling with sensitive characteristic: design and analysis Metrika 2008 67 3 251 263 10.1007/s00184-007-0131-x

[B9] Warner SL Randomized-response: a survey technique for eliminating evasive answer bias J Am Stat Assoc 1965 60 63 69 10.1080/01621459.1965.10480775 12261830

[B10] Mousavi SA Keramat A Vakilian K Interpretation of opposite-sex friendship based on social ecology model in Iranian female IJPBS 2012 6 2 69 78 24644485 PMC3940019

[B11] Family Health International Behavior surveillance survey in Maharashtra Study conducted by ORG center for social research (ORG CSR) with technical assistance from family health international 2001 New Delhi

[B12] Eaton D Kann L Kinchen S Shanklin S Ross J Hawkins J Youth risk behavior surveillance--United States, 2007 Morb Mortal Wkly Rep Surveill Summ 2008 57 4 1 131 18528314

[B13] Grunbauma JA Lowry R Kann L Patemanb B Prevalence of health risk behaviors among Asian American/Pacific Islander high school students J Adolesc Health 2000 27 5 322 330 10.1016/S1054-139X(00)00093-8 11044704

[B14] Asadi A Barrasi ravabete beine dokhtar va pesar dar daneshgahaye shahre Ardabil J Ardabil Univ Med Sci 2006 6 2 113 114 Persian

[B15] Ghaffari M Niknami S Mohsen B Kazemnejad A Mirzaee E Ghofranipour F Correlates of the intention to remain sexually inactive among male adolescents in an Islamic Country: case of the Republic of Iran J Sch Health 2009 79 3 123 129 10.1111/j.1746-1561.2008.0396.x 19207518

[B16] L’Engle KL Brown JD Kenneavy K The mass media are an important context for adolescents’ sexual behavior J Adolesc Health 2006 38 3 92 186 10.1016/j.jadohealth.2005.03.020 16488814

[B17] Smith G Kippax S Aggleton P Tyrer P HIV/AIDS school-based education in selected Asia-Pacific countries Sex Edu 2003 3 1 3 21 10.1080/1468181032000052126

[B18] Altman D Global sex 2001 Sydney: Allen & Unwi: Sydney University of Chicago Press

