# Supplementary material for: Estimation of sexual behavior in the 18-to-24-years-old Iranian youth based on a crosswise model study
Source: BMC Res Notes. 2014 Jan 13;7:28. doi: 10.1186/1756-0500-7-28 (PMC3895705; doi:10.1186/1756-0500-7-28)
Supplement: Additional file 1 — Questionnaire. [file 1756-0500-7-28-S1.doc]

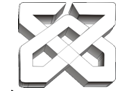


Medical Science Of Shahroud University

Dear participants: Thank you that you participate in this study. This is PhD thesis the to assess the reproductive health needs of Iranian young people. I hope you trust me and answer honestly. answer.

First section

Demographic Variables( some variables)

1. Age
2. Sex
3. Residential area
4. Marriage status
5. Father education
6. Mother education
7. Did you pass life skill?
8. Did you passed Family planning course?
9. Did you go away from home?
10. Did you use pornography media in the 6 month past?
11. What kind media did you use ?

Second section :

Dear participant : For questions that are personal and may not be clearly likes people respond to them below are brought some questions that are designed to double that never answered the sensitive questions for one individual is not detectable.

Guidance Note There are two choices A and B below the questions. Participants tick the square A if their answer to both questions is similar (both Yes or both No) and square B if their answer to one question is Yes and to the other question is No.

| Please consider a male friend or family :  do you have any friend or relative named Ali or Mohammad?” | Set of question |
| --- | --- |
| **A:Both of them are" Yes" or** "No"  **B:One is 'No" and other is "yes"** | Answer |
