# Supplementary material for: Estimation of sexual behavior in the 18-to-24-years-old Iranian youth based on a crosswise model study
Source: BMC Res Notes. 2014 Jan 13;7:28. doi: 10.1186/1756-0500-7-28 (PMC3895705; doi:10.1186/1756-0500-7-28)
Supplement: Additional file 2 — Questionnaire research project. [file 1756-0500-7-28-S2.pdf]

In The Name Of God

Medical Science of Shahroud University

Questionnaire research project

Name and Family name: Katayon Vakilian

Advisor professor:Dr, Seyed Abbas Mousavi,Dr.Afsaneh Keramat

Counseling professor:Dr.Reza Chaman

Incorporated Organization: Medical science Shahroud university

:

Title proposal : Development, Adaptation Iranian youth reproductive health instrument and survey of its situation in Shahroud universities in 1390

Sagessted date: 2010,2.12

Address :Squer Hafte Tir.Medical Science Shahroud University,Shahroud ,Iran
